# The Invasive Plant *Alternanthera philoxeroides* Was Suppressed More Intensively than Its Native Congener by a Native Generalist: Implications for the Biotic Resistance Hypothesis

**DOI:** 10.1371/journal.pone.0083619

**Published:** 2013-12-26

**Authors:** Shufeng Fan, Dan Yu, Chunhua Liu

**Affiliations:** The National Field Station of Freshwater Ecosystem of Liangzi Lake, College of Life Science, Wuhan University, Wuhan, P.R. China; University of Saskatchewan, Canada

## Abstract

Prior studies on preferences of native herbivores for native or exotic plants have tested both the enemy release hypothesis and the biotic resistance hypothesis and have reported inconsistent results. The different levels of resistance of native and exotic plants to native herbivores could resolve this controversy, but little attention has been paid to this issue. In this study, we investigated population performance, photosynthesis, leaf nitrogen concentration, and the constitutive and induced resistances of the successful invasive plant, *Alternanthera philoxeroides*, and its native congener, *Alternanthera sessilis*, in the presence of three population densities of the grasshopper, *Atractomorpha sinensis*. When the grasshopper was absent, leaf biomass, total biomass, photosynthesis, and leaf nitrogen concentration of *A. philoxeroides* were higher than those of *A. sessilis*. However, the morphological and physiological performances of *A. philoxeroides* were all decreased more intensively than *A. sessilis* after herbivory by grasshoppers. Especially as the concentrations of constitutive lignin and cellulose in leaf of *A. philoxeroides* were higher than *A. sessilis*, *A. philoxeroides* exhibited increased leaf lignin concentration to reduce its palatability only at severe herbivore load, whereas, leaf lignin, cellulose, and polyphenolic concentrations of *A. sessilis* all increased with increasing herbivory pressure, and cellulose and polyphenolic concentrations were higher in *A. sessilis* than in *A. philoxeroides* after herbivory. Our study indicated that the capability of the invasive plant to respond to native insect damage was lower than the native plant, and the invasive plant was suppressed more intensively than its native congener by the native insect. Our results support the biotic resistance hypothesis and suggest that native herbivores can constrain the abundance and reduce the adverse effects of invasive species.

## Introduction

Invasive species cause significant ecological and socio-economic effects in introduced areas. To understand the mechanisms that allow exotics to become invasive, many hypotheses have been proposed. The enemy release hypothesis predicts that, in the absence of specialist enemies, generalist enemies have a greater impact on native competitors, which allows exotic species to outperform natives in introduced regions [1]. In contrast, the biotic resistance hypothesis proposes that native enemies prefer exotic species over native species. The exotic species share no evolutionary history with native enemies and, hence, lack effective defenses against them [2].

Many studies have tested these two hypotheses in different plant-animal systems, but results have been inconsistent. For instance, some studies have found that exotic plants suffered less herbivory than native plants in introduced ranges [3]–[6], which is consistent with the enemy release hypothesis. However, other studies have shown that native herbivores prefer exotic plants over native plants [2], [7]–[9], which supports the biotic resistance hypothesis. Even within the same plant-animal system, conflicting results have been reported, depending on the field and laboratory settings. For example, Zas et al. [10] found that pine weevils obviously preferred native pines over exotic pines in Petri dishes; however, this result was opposite in field trials because native pines produced more induced resistance than exotic congeners.

Herbivores affect plant growth and fitness not only by damaging organs and tissues (e.g., leaves, phloem, roots, and twigs) but also by altering physiological traits. For instance, herbivory often affects the concentrations of available nitrogen and other important nutrients in foliage [11], significantly decreases photosynthetic activity [12], [13], and increases leaf conductance, transpiration rate, and intercellular CO_2_ concentration [14] of the remaining intact tissue. To minimize damage, plants have developed resistance strategies against herbivores. Resistance strategies should reduce the preference or performance of herbivores and include constitutive resistance (permanently expressed irrespective of herbivore attacks) and induced resistance (expressed only after herbivore attacks) [15]. For instance, when an herbivore attacks a plant, phytohormone ethylene is produced by the damaged tissue and may influence the production of phenylalanine ammonia lyase, which determines the production of phenolics (such as lignin and other secondary metabolites), and ultimately affects leaf toughness [11]. In addition, this induced resistance could grow as the level of damage to the plant increases [11], [16].

In introduced ranges, except when specialists switch to exotic congeners or specialists of exotic plants also are introduced to the same area, most exotic plants are free from specialist attack [1], but they suffer damage from generalists, as native plants do. Theoretically, congeneric plants have similar growth and defense strategies [17], [18]. However, according to the biotic resistance hypothesis, native plants should be able to defend against native generalist attack more effectively than exotics. Alternatively, if exotic plant defenses are uncommon or absent in the introduced range, native generalists would be deterred due to a lack of an effective detoxification mechanism [3], [19]. Therefore, differences in defenses between native and exotic species may explain native herbivore preference for them [10], however, little attention has been paid to this aspect in the literature. Pearsea and Andrew [20] found the similarity of defensive traits between exotic and the native oak was predictive of the degree of chewing-guild herbivory that exotic oaks suffered. Zas et al. [10] found that the native large pine weevil, *Hylobius abietis*, preferred the exotic pine, *Pinus radiata,* over its native congener, *P. pinaster*, presumably because the native pine produced more resin in the stems when attacked. This study concluded that the native herbivore played a role in preventing *P. radiata* from invading the region [10]. Carrillo-Gavilán et al. [21] also observed that the total amount of phenolics induced by herbivory damage from native, generalist insects were significantly greater in the native pines than in the closely related exotic pines. In addition, the concentration of total constitutive phenolics was higher in needles of exotic pines and in stems of native pines.

The study subjects mentioned above were all exotic non-invasive plants. If an exotic plant is prevented from becoming invasive because it is less effective at resisting herbivory from native generalists than the native plant, it would be logical to assume that a successful invasive plant may be more effective at defending against native generalists than the native plant. However, we currently know little about differences in resistance strategies against native herbivores between native and successful invasive plants. Previous study showed biochemistry of invasive plant is no more deterrent to a native generalist herbivore than extracts from native plants [22], but the study did not relate to induced resistance. In this study, we investigated damage caused by a native generalist grasshopper, *Atractomorpha sinensis*, to an invasive aquatic plant, *Alternanthera philoxeroides*, and its native congener, *Alternanthera sessilis*. Performance and physiological responses, resistance strategies, and resistance intensity of the two plants to three population densities of grasshoppers were also evaluated. We attempted to address the following questions: (i) did the invasive plant suffer less damage than its native congener? (ii) were there any differences in performance and physiological responses in the two plants when they were attacked by *A*. *sinensis*? (iii) was the invasive plant more effective at defending against the native generalist than the native plant? (iv) were there any differences in resistance strategies of the plants at different levels of herbivore load?

## Materials and Methods

### Study Materials

*Alternanthera philoxeroides* (Martius) Grisebach (Amaranthaceae), commonly known as alligator weed, is a perennial herbaceous plant that is both stoloniferous and amphibious. It can grow prostrate along the ground or across the water surface, rooting at the nodes, anchoring to the shore, and forming tangled mats. The native range of this species is thought to be the Parana River region of southern Brazil, Paraguay, and Argentina. It can also be found in coastal Brazil and northern areas of South America [23]. Currently, *A. philoxeroides* has invaded the USA, China, Australia, New Zealand, Indonesia, India, and Thailand [24] and has caused economic and ecological problems in these regions. In China, *A. philoxeroides* was first introduced into suburban Shanghai from Japan as a forage crop in the late 1930s. It was then spread intentionally to eastern and southern China between the 1950s and the 1970s, has now invaded large areas south of the Yellow River Basin, and can be found sporadically in northern China. *A. philoxeroides* has been listed as one of the 12 most harmful alien, invasive species in China [25]. In its native range, *A. philoxeroides* has many parasitic natural enemies. These enemies, especially specialists feeding on different organs and tissues of *A. philoxeroides*, regulate its population [26]–[28]. In China, more than ten generalist insects feed on *A. philoxeroides* [29], [30]. However, no literature was found discussing either the preference of these generalist insects for *A. philoxeroides* versus native plants or the defense mechanisms of *A. philoxeroides* and native plants against these generalists.

*Alternanthera sessilis* (L.) DC. is native to China and is also a stoloniferous and amphibious perennial herbaceous plant. Similar to *A. philoxeroides*, it generally grows on roadsides, in gardens, in swamps, and in streams and has the ability to grow prostrate along the ground, the shore, or float on water, rooting at the nodes. However, unlike *A. philoxeroides*, it cannot form tangled mats on the surface of a water body. *A. sessilis* occurs in Bhutan, Cambodia, India, Indonesia, Laos, Malaysia, Myanmar, Nepal, Philippines, Sikkim, Thailand, and Vietnam. In China, it is distributed in most of the provinces south of the Yellow River [31]. Because *A. sessilis* shares the same phylogenetic history and has similar morphological traits and habitats to *A. philoxeroides*, it has often been used for comparisons with *A. philoxeroides* in studies on invasion mechanisms [32], [33].

*Atractomorpha sinensis* Bolivar is a ubiquitous generalist grasshopper native to China. It primarily feeds on dicotyledonous plants, causing damage to a large number of vegetables, crops, and grasses. In field investigations we found *A. sinensis* also fed on the leaves of *A. philoxeroides* and *A. sessilis*.

### Ethics Statement

Plants material was collected from natural populations at the National Field Station of Freshwater Ecosystem at Liangzi Lake. All larvae of *A. sinensis* were collected from grass at the National Field Station of Freshwater Ecosystem at Liangzi Lake and starved for one day before the experiment. All grasshoppers were released after the experiment was completed.

### Experimental Design

This experiment was conducted at the National Field Station of Freshwater Ecosystem at Liangzi Lake, Hubei Province, China (30°50′–30°180′N, 114°210′–114°390′E). On April 23, 2012, 94 shoots of *A. sessilis* and 77 shoots of *A. philoxeroide* were cultivated in circular basins with sandy sediment and 5 cm of water. All shoots were approximately 10 cm long, with two nodes and three pairs of leaves. One week later, 36 plants of each species with similar height and weight (the mean heights were 13.21±1.09 cm and 12.71±1.02 cm; the mean fresh weights were 1.89±0.26 g and 1.00±0.16 g; and the mean lengths of roots were 3.4±0.65 cm and 4.3±0.84 cm for *A. philoxeroides* and for *A. sessilis,* respectively) were transferred to 36 aquaria (100 cm length × 50 cm width × 70 cm height) that were filled with 15 cm of fine-textured, homogeneous sediment soil. Two plants of one species were planted in each aquarium and all aquaria were placed on an outdoor, cement platform. The experimental systems were maintained daily, and the soil was saturated with water.

After 16 weeks, the two plants in each aquarium developed into a single population and all leaves were intact, with no herbivore bite marks. The population densities of *A. philoxeroides* and *A. sessilis* were 29.5±5.5 and 35.3±9.6 plants per square meter, respectively. Each aquarium was randomly assigned to one of three treatments- mild herbivory, severe herbivory, or control. Each species-treatment combination had six replicates. In the mild herbivore load group, we randomly picked out six aquaria of each species and put seven larvae of *A. sinensis* in each aquarium. In the severe herbivore load group, six aquaria of each species were picked out randomly and each aquarium received twenty larvae of *A. sinensis*. The last six aquaria of each species were used as controls. All aquaria were covered by white nylon web (mesh size: 1 mm^2^) throughout the experiment.

After 19 days, almost all leaves of the two species under severe herbivore load were gnawed by *A. sinensis*, but the stems of the two species under both mild and severe herbivore load were still intact. Control plants had no herbivore bite marks. The net photosynthetic rate was determined using a Li-6400 Portable Photosynthesis System (Li-Cor, USA) under a photosynthetic photon flux density (PPFD) of natural light >1700 µmol m^–2^ s^–1^ for either the second or the third pair of leaves from the top of each plant. The air temperature was moderate (25–30°C) and the relative humidity ranged from 60 to 70% between the hours of 11∶00 and 14∶00. We measured net photosynthetic rate on a single, undamaged leaf in each control aquarium and one undamaged and one herbivory damaged leaf in each mild herbivory treatment aquarium. In the severe herbivory treatment, we only measured damaged leaves because there were very few undamaged leaves. The leaves used for the photosynthesis measurements were marked and used for measuring the maximal quantum yield (Fv/Fm) using a DIVING-PAM (WALZ, Germany) between 20∶30 and 21∶00. Next, these leaves were detached, and the leaf area was measured using a Li-3100 Area Meter (Li-Cor, USA) to calculate the light-saturated photosynthetic rate per unit leaf area (P_max_).

The next day, all grasshoppers were removed. All second and third pairs of leaves from the top of each plant were detached, dried at 70°C for more than 48 h, and stored at –20°C for chemical analyses. Lastly, the plants were harvested; the leaves, stems, and roots were separated, washed, and dried at 70°C for more than 48 h to determine the leaf biomass and total biomass of each population. Leaf nitrogen concentration based on mass (N_mass_) was determined using an element analyzer, Euro EA3000 (Euro Vector, Italy). Leaf polyphenolic concentrations were determined by the Folin-Ciocalteau method [34]. Leaf lignin content was determined using the method by Biqinluoke [35]. Leaf cellulose content was determined by anthrone colourimetry [36].

### Statistical Analyses

All data including total biomass, leaf biomass, P_max_, Fv/Fm, and leaf nitrogen, lignin, cellulose and polyphenolic concentrations within the three herbivory treatments (except these traits for undamaged leaves at the mild herbivore load level) were analyzed with a factorial ANOVA assuming all effects (species, herbivory, and their interaction) as fixed factors, after testing for normality and homoscedasticity. Duncan tests were used to compare levels within factors for significance (P<0.05). The differences in P_max_, Fv/Fm, and leaf nitrogen, lignin, cellulose and polyphenolic concentrations between damage and undamaged leaves under mild herbivore load were analyzed using a paired T Test. Polyphenolic concentration data were transformed using a Sqrt (x) function. All analyses were performed using SPSS 13.0 (SPSS Inc., Chicago, IL, USA).

## Results

### Plant Performance

**Figure 1:**
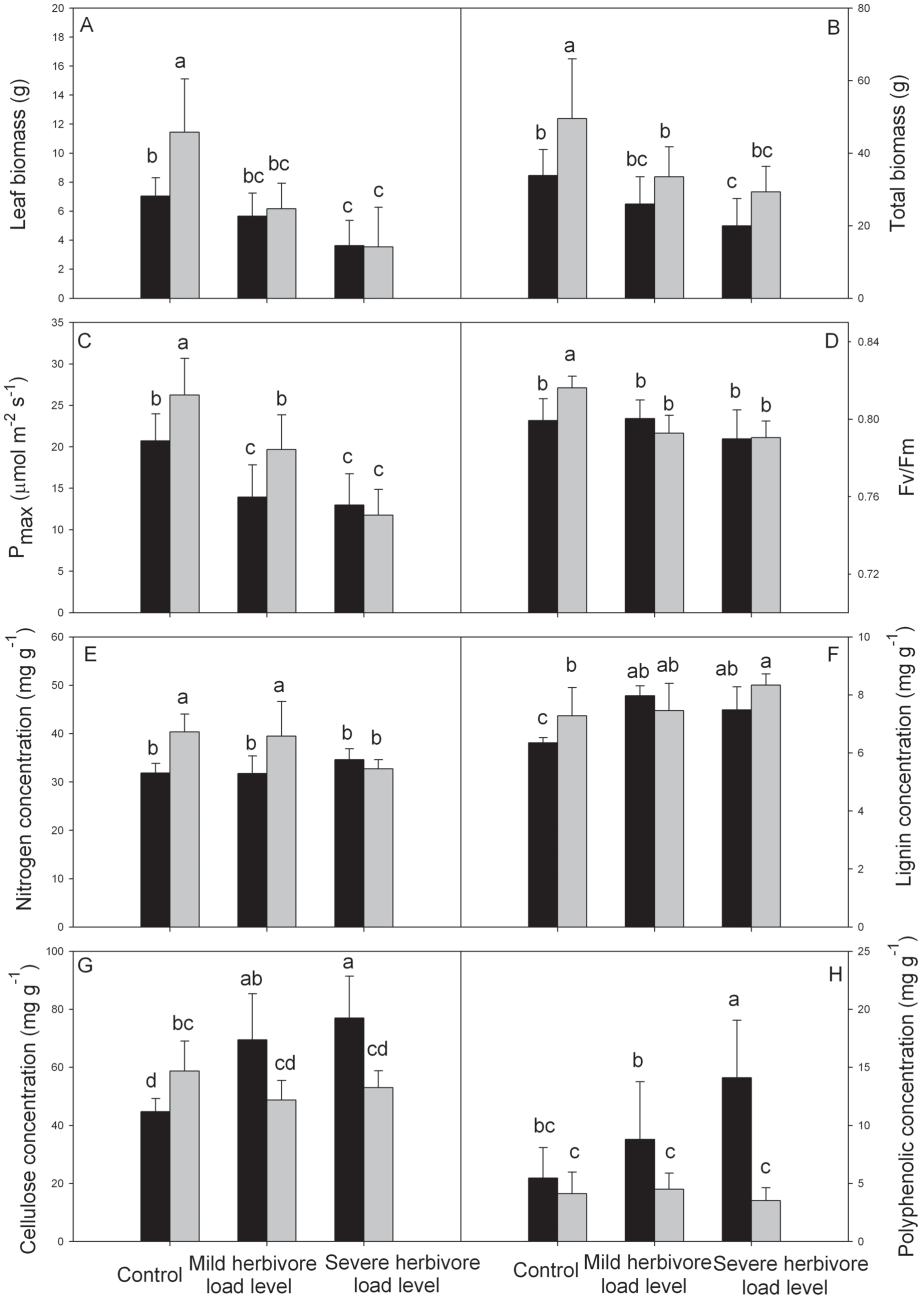
More damage and weaker resistance in invasive plant after herbivory. Differences of leaf biomass (a), total biomass (b), P_max_ (c), Fv/Fm (d), leaf nitrogen concentration (e), leaf lignin concentration (f), leaf cellulose concentration (g) and leaf polyphenolic concentration (h) (mean ±SD) between *A. sessilis* (black bars) and *A. philoxeroides* (grey bars) at three herbivore load levels.

Leaf biomass and total biomass showed an obvious decrease with increasing herbivore load levels (Table 1, Fig. 1a, b). A significant species×herbivory interaction indicated that changes in leaf biomass after herbivory differed among the two species (Table 1). Although leaf biomass of *A. philoxeroides* was higher than *A. sessilis* in the control group, it was similar between the two species at both mild and severe herbivore load levels (Fig. 1a), due to sharper decreases in *A. philoxeroides* at both herbivore load levels (leaf biomass of *A. philoxeroides* decreased 46.2% and 69.2% at mild and severe herbivore load levels, respectively, while the decreases in *A. sessilis* were 19.8% and 48.7% at mild and severe herbivore load levels, respectively). Total biomass of *A. philoxeroides* was significantly higher than that of *A. sessilis* in the control group, although the differences were weakened at both mild and severe herbivore load levels, species×herbivory interaction was non-significant, total biomass of *A. philoxeroides* were still higher than *A. sessilis* (Table 1, Fig. 1b).

**Table 1:** *F* and *P* values of the leaf biomass, total biomass, P_max_, Fv/Fm, N concentration, lignin concentration, cellulose concentration and polyphenolic concentration for the two species and three herbivore load levels (except these traits for undamaged leaves at mild herbivore load level) calculated using a factorial ANOVA.

Source		d.f.	F value	P value
Leaf mass	Species	1,6	4.496	**0.042**
	Herbivory	2,6	18.755	**<0.001**
	Species×Herbivory	2,6	3.415	**0.046**
Total biomass	Species	1,6	11.518	**0.002**
	Herbivory	2,6	10.033	**<0.001**
	Species×Herbivory	2,6	0.607	0.551(ns)
P_max_	Species	1,6	6.930	**0.013**
	Herbivory	2,6	25.890	**<0.001**
	Species×Herbivory	2,6	3.223	**0.054**
Fv/Fm	Species	1,6	0.938	0.341(ns)
	Herbivory	2,6	8.911	**0.001**
	Species×Herbivory	2,6	4.315	**0.023**
N concentration	Species	1,6	13.456	**0.001**
	Herbivory	2,6	1.303	0.287(ns)
	Species×Herbivory	2,6	6.664	**0.004**
Lignin concentration	Species	1,6	3.061	0.092(ns)
	Herbivory	2,6	7.799	**0.002**
	Species×Herbivory	2,6	3.756	**0.036**
Cellulose concentration	Species	1,6	8.484	**0.007**
	Herbivory	2,6	4.771	**0.016**
	Species×Herbivory	2,6	11.961	**<0.001**
Polyphenol concentration	Species	1,6	24.549	**<0.001**
	Herbivory	2,6	4.632	**0.018**
	Species×Herbivory	2,6	6.422	**0.005**

Boldface denotes significance, ns denotes no significance.

**Figure 2:**
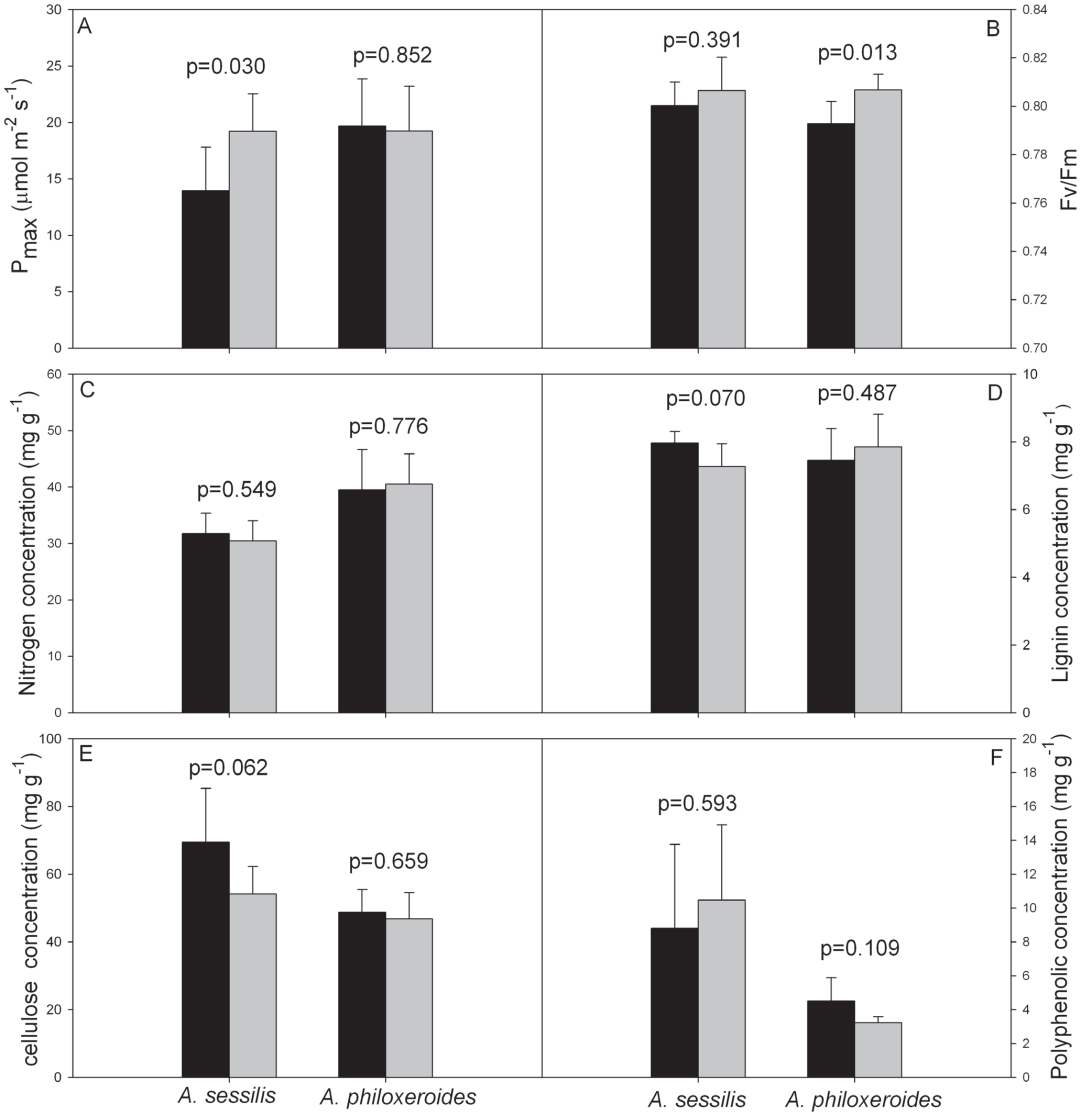
Undamaged leaf of damaged naive plants don’t decrease photosynthesis or produce defense compounds. Differences of P_max_ (a), Fv/Fm (b), leaf nitrogen (c) lignin (d), cellulose (e) and polyphenolic (f) concentrations between damaged (black bars) and undamaged leaves (grey bars) of the two plant species.

Herbivory significantly decreased the P_max_, Fv/Fm, and leaf nitrogen concentration of *A. philoxeroides*, but it only decreased the P_max_ of *A. sessilis* (Table 1, Fig. 1c, d, e). Changes to Fv/Fm, and leaf nitrogen concentration after herbivory differed among the two species, as indicated by the significant species×herbivory interactions (Table 1). In control groups, the Fv/Fm, and leaf nitrogen concentration of *A. philoxeroides* were higher than *A. sessilis*, however, these differences all disappeared at severe herbivore load levels (Fig. 1d, e). Although the species×herbivory interaction for P_max_ was not as strong as interactions for Fv/Fm, and leaf nitrogen concentration, it was moderately significant (p = 0.054), especially the sample size was low. And similar to Fv/Fm, and leaf nitrogen concentration, P_max_ of *A. philoxeroides* was higher than *A. sessilis* in control group, but difference also disappeared at severe herbivore load level (Fig. 1c). In addition, under mild herbivore load, the P_max_ of undamaged *A. sessilis* leaves was significantly higher than damaged leaves, but there was no difference between damaged and undamaged leaves of *A. philoxeroides* (Fig. 2a). Conversely, the Fv/Fm of undamaged leaves of *A. philoxeroides* was significantly higher than damaged leaves, but damaged and undamaged leaves of *A. sessilis* had similar Fv/Fm (Fig. 2b).

### Plant Defense

Herbivory greatly increased the leaf lignin, cellulose, and polyphenolic concentrations of *A. sessilis*, but it only increased the leaf lignin concentration of *A. philoxeroides* (Table 1, Fig. 1f, g, h). The induction of these traits after herbivory differed among the two species, as indicated by the significant species×herbivory interactions (Table 1). The leaf lignin concentration of *A. philoxeroides* was higher than *A. sessilis* in the control group, but the two species had similar leaf lignin concentrations after herbivory by *A. sinensis* (Fig. 1f). In the control group, the leaf cellulose concentration of *A. philoxeroides* was significantly higher than that of *A. sessilis*, but the differences were reversed at both mild and severe herbivore load levels, due to the increase in leaf cellulose concentration in *A. sessilis* (Fig. 1g). Although the two species had similar leaf polyphenolic concentrations in the control group, *A. sessilis* exhibited significantly higher leaf polyphenolic level when compared to *A. philoxeroides* at both mild and severe herbivore load levels (Fig. 1h). In addition, under mild herbivore load, while not significant at the alpha = 0.05 level, there was strong evidence suggesting that lignin and cellulose concentrations in undamaged leaves of *A. sessilis* were lower than in damaged leaves (p = 0.07 and p = 0.062), but these traits did not differ in damaged and undamaged leaves of *A. philoxeroides* (Fig. 2d, e).

## Discussion

The biotic resistance hypothesis proposes that native enemies have a greater impact on exotic plants than on native plants [2]. Consistent with this hypothesis, our data indicated that both morphological traits (biomass and leaf biomass) and physiological traits (P_max,_ Fv/Fm and leaf N concentration) of the invasive plant *A. philoxeroides* were suppressed more intensely than those in its native congener *A. sessilis,* by the native generalist *A. sinensis*.

Under the same population densities of *A. sinensis*, more leaves of *A. philoxeroides* were consumed than *A. sessilis*. Therefore, more leaf biomass (hence the population biomass) and a larger leaf area for photosynthesis were reduced when compared to *A. sessilis*. Furthermore, Zangerl et al. [12] proposed that the indirect impact of reduced photosynthesis (due to herbivory pressure) on the loss of plant population biomass was greater than the direct impact of herbivores on the loss of biomass. This study found that caterpillar feeding remarkably decreased photosynthesis of the remaining, intact leaf tissue, as measured by both gas exchange and fluorescence imaging. Consistent with previous studies [12]–[14], we also found that compared with controls, P_max_ of the remaining, intact leaves of the two species decreased significantly. However, Fv/Fm of the remaining, intact leaves decreased significantly only in *A. philoxeroides*. Moreover, our data reveal that the photosynthetic capacity of *A. philoxeroides* was more intensely suppressed than that of *A. sessilis* in the presence of the grasshopper, which may explain why the population biomass of *A. philoxeroides* was reduced more than *A. sessilis* when herbivores were present. The leaf N concentration of *A. philoxeroides* decreased significantly at severe herbivore load, but leaf N concentration of *A. sessilis* was not affected by herbivory. Because proteins participate in the Calvin cycle and represent the majority of the leaf nitrogen content, leaf photosynthesis correlates positively with protein content, hence the leaf nitrogen content [37]. Although lower nitrogen concentrations can decrease photosynthetic ability and relative growth rate, it is also associated with low palatability and has been suggested as one anti-herbivore strategy for plants [38]–[40].

Lignin and cellulose are major components of plant cell wall. Elevating lignin and cellulose contents increases leaf toughness and reduces plant palatability [41]. Polyphenol is a quantitative defensive component of plant quality, and has negative effects on the development and reproduction of herbivorous insects [42]. In this study, we found that concentrations of constitutive lignin and cellulose in leaf of *A. philoxeroides* were higher than *A. sessilis*, both species had similar levels of leaf polyphenol. However, leaf polyphenolic, lignin, and cellulose concentrations all increased in *A. sessilis* after herbivory by grasshoppers. In contrast, only the leaf lignin concentration increased in *A. philoxeroides* after herbivory. In addition, the concentrations of cellulose and polyphenol in *A. sessilis* were higher than in *A. philoxeroides* when grasshoppers were present. The presence of polyphenol can affect food choices in some grasshoppers [43]. Recent research also found the leaves of a plant population with lower tannin content were consumed by caterpillars more than those with higher tannin content, and caterpillar performance was higher on leaves with lower tannin content [44]. Therefore, we suggest that elevated defense compounds in *A. sessilis* decrease grasshopper performance, resulting in less leaf loss in *A. sessilis*. Note that when the grasshopper population was low, the lignin and cellulose concentrations in undamaged leaves of *A. sessilis* were lower than damaged leaves. Implying that in damaged plants of *A. sessilis*, a leaf did not produce defense compounds until it was chewed by grasshoppers. Müller-Schärer et al. [45] noted that high level of lignin reduced not only leaf palatability but also specific leaf area (SLA), one of the main determinants of the relative growth rate. In this study, we also found that leaf lignin concentration was negatively correlated with P_max_ (R = 0.306, P<0.05). Therefore, producing more lignin and cellulose might result in the growth rate of the whole plant decreasing more sharply. We propose that *A. sessilis* might have adapted a strategy in which it can defend against herbivore, and reduce growth rate of itself as little as possible when the threat of herbivory is low.

Our results were consistent with the findings in Zas et al. [10], which found that, compared to invasive plants, the capability of the native congener to respond to native insect damage was stronger. The weaker induced resistance of *A. philoxeroides* can contribute to its impaired competitiveness with *A. sessilis* in the presence of the native generalist grasshoppers. Because our study was conducted in a closed system in which grasshoppers could not move freely to plants with lower induced resistance and higher palatability, we expect that the consequences would be further magnified in the field, where the two plants co-exist and where herbivores are not restricted.

Exotic prey usually lack effective defenses against native enemies in new regions where they share no evolutionary history with those enemies and have not experienced selection from them; therefore, native consumers may prefer exotic over native prey [2]. When exotic species encounter a novel, non-coevolved enemy, toxin-based defenses plants could obtain an inherent advantage because novel enemies lack proper detoxification mechanisms to unknown toxins, which would contribute to enemy release and invasive spread. However, elicitor-receptor plants would fail to recognize novel, non-coevolved enemies, which would contribute to biotic resistance and suppression of the invasion [46]. In our study, the invasive plant *A. philoxeroides* failed to respond to the attack of novel, non-coevolved enemies. In its original native range, the primary regulators of *A. philoxeroides* are specialists [26]–[28], which may exclude generalists from preying on the plants. The defense strategy of *A. philoxeroides* may aim primarily at specialists and therefore may be less efficient at deterring generalists. In our study, the induced resistance of *A. philoxeroides* was worse while its constitutive resistance was better when compared to its native congener, *A. sessilis*. Therefore, as the biotic resistance hypothesis suggests, our results show that the native generalist *A. sinensis* can control the population of the invasive plant *A. philoxeroides* more efficiently than its native congener *A. sessilis*, and thus potentially limits the invasion of the exotic plant [47].

In summary, we found that the induced resistance of *A. sessilis* was more efficient and sophisticated than that of the invasive congener *A. philoxeroides*. Population performance, photosynthetic capacity and leaf nutrition content were reduced more in the invasive plant when compared to the metrics in the native plant. Our study suggests that the native herbivores can suppress the invasive plants. Although they rarely resist an invasion completely, native herbivores can constrain the abundance and reduce the adverse effects of invasive species once they have successfully established [48].
